# Evaluation of an Indirect-ELISA Test for *Trypanosoma evansi* Infection (Surra) in Buffaloes and Its Application to a Serological Survey in Thailand

**DOI:** 10.1155/2015/361037

**Published:** 2015-05-25

**Authors:** Arthur Kocher, Marc Desquesnes, Ketsarin Kamyingkird, Sarawut Yangtara, Emilye Leboucher, Pranee Rodtian, Alan Dargantes, Sathaporn Jittapalapong

**Affiliations:** ^1^Centre de Coopération Internationale en Recherche Agronomique pour le Développement (CIRAD), UMR InterTryp, 34398 Montpellier, France; ^2^Faculty of Veterinary Medicine, Kasetsart University, Chatuchak, Bangkok 10900, Thailand; ^3^The Fifth Regional Livestock Office, Department of Livestock Development (DLD), Chiang Mai 50300, Thailand; ^4^Central Mindanao University, 8710 Mindanao, Philippines

## Abstract

Surra, caused by *Trypanosoma evansi*, is a neglected disease due to frequent subclinical evolution, especially in bovines in Asia. However, acute and chronic signs are regularly observed, with significant sanitary and economic impacts. In this study, we evaluated and applied an indirect-ELISA test for the detection of anti-*T. evansi* immunoglobulin G in buffaloes using antibovine conjugate. Based on buffalo reference sera from the Philippines, a two-graph receiver operating characteristics analysis (TG-ROC) was conducted to define an optimal cut-off value; sensitivity and specificity were estimated at 92.5% and 94.2%, respectively. A cross-sectional serological survey was carried out in the major buffalo breeding areas of Thailand; 892 buffaloes from 8 provinces were sampled in North, Northeastern, and Southern Thailand. Seropositive buffaloes were found in all 8 provinces, on 20.3% of farms for an overall prevalence of 12.2% (95% CI 10.2–14.5%). Nearly one-third of the sampled population was exposed to infection. Broader sampling would be necessary but is not possible in the southern half-wild breeding systems. According to our results, buffaloes may constitute a large and robust reservoir for *T. evansi*, which is a permanent threat to other livestock such as cattle and horses as well as wild animals such as elephants in Southest Asia.

## 1. Introduction

Surra is a multispecies disease caused by* Trypanosoma evansi*, originating from Africa, where it mainly affects camels. It spread to Latin America and Asia, where it mostly affects horses, dogs, cattle, and buffaloes [1]. It is an underestimated and neglected disease because of the frequent subclinical evolution of the infection. This is especially the case in Asian swamp buffaloes, because, even though clinical signs are frequent at the moment of the outbreak, most of the animals become healthy carriers after some time and may act as reservoirs [2]. These healthy carriers may relapse into clinical infection under the pressure of stress or intercurrent diseases and thus remain permanent sources of infection for susceptible livestock, leading to enzo-epizootic situations [3]. Under these conditions, the sensitivity of parasitological tests is very low and it is difficult to establish a clear diagnosis. Consequently, assessing the sanitary and economic impacts of surra is even more challenging. However, recent studies based on population modelling demonstrated a highly significant impact of surra in buffaloes in the Philippines [4]. Other studies demonstrated the interference of surra in vaccination against Foot and Mouth Disease (FMD) [5, 6] and haemorrhagic septicaemia (HS) [7, 8], as suggested from field observations [9]. In the context of a large-scale control campaign against FMD and HS in Southeast Asia where surra is enzootic, this draws fresh attention to the disease.

Antibody detection using indirect-ELISA with soluble antigens of* T. evansi* from whole cell lysate is recommended by the OIE [10] and has been used for a long time [11]. Standardization and validation methods have been developed [12–15]. ELISA* T. evansi*, initially used on camels [16], has been adapted to other species such as horses [17, 18], buffaloes [19], cattle [20], and pigs [21]. However, the local development of serological tests, including parasite production, mean population responses, and selection of control sera is necessary to cope with the variability in response that may exist between parasite stocks and hosts from different geographic areas [13, 22].

A survey carried out in Thai dairy cattle demonstrated the presence of* T. evansi* infection in 12 out of the 13 provinces investigated and showed that around 25% of the cattle population was exposed to the infection [23]. Little is known on the potential impact of surra in the Thai buffalo population which was estimated at around 1.4 million head in 2009. Here, we standardized and evaluated an indirect-ELISA test for the detection of anti-*T. evansi* immunoglobulins G (IgG) in buffaloes. The test was then applied to a serological survey in the main buffalo-breeding areas of Thailand.

## 2. Materials and Methods

### 2.1. Indirect-ELISA* T. evansi* Standardization

#### 2.1.1. Protocol

The preparation of soluble antigens from a* T. evansi* strain isolated in Thailand and ELISA procedures were derived from a study previously carried out in dairy cattle in Thailand [23].

In preliminary studies, performance of ELISA in buffaloes was compared between Protein A and antibovine conjugates. The latest provided a higher contrast between samples from infected and noninfected animals. Consequently, antibovine conjugate was used in the present study.

Briefly, Microtest 96-well Polysorp Nunc immunoplates (Nunc, Roskilde, Denmark) were coated with 100 *μ*L/well of* T. evansi* soluble antigen (5 *μ*g/mL) in carbonate buffer (0.05 M, pH 9.6) and incubated overnight at 4°C. The plates were blocked with 150 *μ*L/well of blocking buffer (BB) (Phosphate Saline Buffer (PBS), 0.1% Tween 20 (Labchem, Pittsburg, USA), 7% skim milk powder (ref.: 190-12865, Wako Pure Chemical Industries Ltd., Osaka, Japan)) with permanent shaking (150 rpm) for 30 min at 37°C. The BB was discarded. Sera diluted 1 : 100 in BB were transferred in duplicate to the ELISA plates. After incubating, under the same conditions as previously, the plates were washed eight times with washing buffer (WB) (PBS, 0.1% Tween 20). Then, 100 *μ*L of peroxidase-conjugated antibovine IgG (ref: A5295, Sigma–Aldrich, USA), diluted 1 : 25,000 in BB, was added and the plates incubated for 30 min at 37°C with shaking (150 rpm). After washing eight times with WB, 100 *μ*L of the substrate/chromogen complex 3,3′,5,5′-tetramethylbenzidine (TMB) (SureBlue TMB, KPL, Maryland, USA) was added. The plates were incubated, without shaking, in a dark room for 30 min. Optical density (OD) was measured at 620 nm in an ELISA reader (Dynex Technologies, VA, USA).

#### 2.1.2. Selection of Controls and Definition of a Relative Percentage of Positivity

Our stock of reference serum samples from infected and noninfected buffaloes being from the Philippines and in limited amount, it was used only to determine the cut off value and the test's performances (see Section 2.1.3).

To be representative of the target population, local control serum was selected from Thai buffalo samples. A first batch of buffalo sera from Northeastern Thailand was analysed by ELISA and the results expressed as optical density (OD). Arbitrary and temporary low and high cut-off values (COVl & COVh) were used in order to define negative and positive samples, respectively. A farm was considered as noninfected if all samples exhibited OD < COVl. A pool of negative samples was made with samples from noninfected farms. A pool of positive samples was made with samples exhibiting OD > COVh. The mean OD of negative and positive samples (*μ*n and *μ*p) were computed. Three negative controls (C−) and three positive controls (C+) (low, medium, and high responses), representative of noninfected and infected Thai buffalo populations were selected as follows:

(1) C1−0.9×μn,C2−≈μn,C3−≈1.1×μn,C1+≈0.9×μp,C2+≈μp,C3+≈1.1×μp.

Afterwards, ELISAs were run for all samples (reference and survey) in duplicate with the three C+, the three C−, and 2 blank wells on each plate. The blank OD value was systematically subtracted from the average OD of each sample and the results were expressed as a relative percentage of positivity (RPP) [13] as follows:

(2) $$\begin{array}{c} \text{RPP sample}=\frac{\text{mean OD sample}-\text{mean OD of C}-}{\text{mean OD of C+}-\text{mean OD of C}-}. \end{array}$$

#### 2.1.3. Selection of the Cut-Off Value and Evaluation of the Test

Reference sera were collected from buffaloes from the Philippines (Central Mindanao University). Forty-one adult buffaloes positive for* T. evansi* using Hematocrit Centrifuge technique (HCT [24]) or mouse inoculation technique (MIT) were used as positive reference samples. Thirty-eight buffaloes native and bred in an area where surra has never been detected were used as negative reference samples. All were negative with HCT, MIT, and the Card agglunitation test for trypanosomiasis (CATT/*T. evansi*, [25]).

The indirect-ELISA test was performed on these samples. The normal distribution of their RPP values was checked with a Shapiro-Wilk test using the stats package in *R*. To optimize the cut-off value, we performed a two-graph receiver operating characteristics analysis (TG-ROC, [26]) using the DiagnosisMed package in *R*. The misclassification cost term method was used [27] with an a priori prevalence of 0.2, and a misclassification cost term of 2. Resulting sensitivity (Se) and specificity (Sp) were computed with 95% confidence intervals.

### 2.2. Survey Area and Sampling

**Figure 1:**
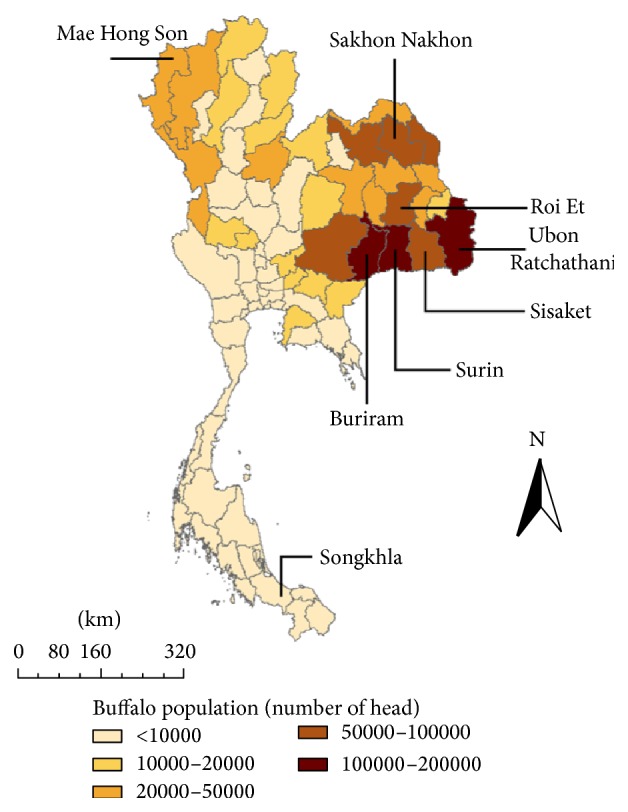
Distribution of buffalo population in Thailand (source: DLD, 2009) and sampling area. Only the names of the provinces sampled are indicated on the map.

Farms were selected randomly among volunteer breeders after they were informed by the Department of Livestock Development (DLD) officers. The study area comprised eight provinces: (i) six provinces of Northeastern Thailand, with the highest buffalo population and where animals are bred in small units (2–4 heads), (ii) one province of Northern Thailand, characterized by very close breeding conditions for cattle and buffaloes, and (iii) one province of Southern Thailand, mostly characterized by a population of medium-sized herds of domesticated buffaloes, and a large population of extensive herds (almost wild animals). These provinces hold 635,000 heads, thus approximately 46% of Thai buffaloes (DLD, 1984–2009; Figure 1, Table 1).

Blood samples were collected in plain and EDTA tubes by jugular venipuncture. When immediate laboratory work was possible, the EDTA tubes were placed inside a cool box pending blood centrifugation in dry capillary tubes (Hirschmann Laborgeräte, Eberstadt, Germany) for packed cell volume (PCV) measurement and microscopic examination for the detection of trypanosomes (HCT, [24]). Blood collected in plain tubes was allowed to clot for 1–4 hours at ambient temperature and then stored at 1–4°C for 24 hours until the serum was separated by centrifugation (500 g) and collected for serological tests.

### 2.3. Data Analysis

Prevalence estimations and confidence intervals computations were made using the epitools webserver (http://epitools.ausvet.com/; [28]). Apparent prevalence values were inferred directly from the indirect-ELISA results and the Wilson binomial approximation [29] was used to compute the confidence limits. True prevalence values and confidence intervals were estimated based on the indirect-ELISA results and the quality of the test (Se and Sp), as previously assessed, using Blaker's method [30]. An infected farm was defined as a farm in which at least one buffalo was positive. The prevalence of infected farms (PF) and the mean prevalence in infected farms (PiF) were computed. All statistical tests were made using *R*.

## 3. Results

### 3.1. ELISA* T. evansi* Standardization

From the first batch of samples from Thailand that were tested in ELISA, a pool of 526 samples was constituted from farms considered as noninfected (all samples exhibiting OD < 0.150). Their mean OD was 0.062. The 3 selected negative controls (C1−, C2−, and C3−) exhibited OD values of 0.054, 0.062, and 0.066, respectively.

Another pool of 16 positive samples was made with samples exhibiting an OD > 0.350; their mean OD was 0.401. The 3 selected positive controls (C1+, C2+, and C3+) exhibited OD values of 0.361, 0.401, and 0.442, respectively.

**Figure 2:**
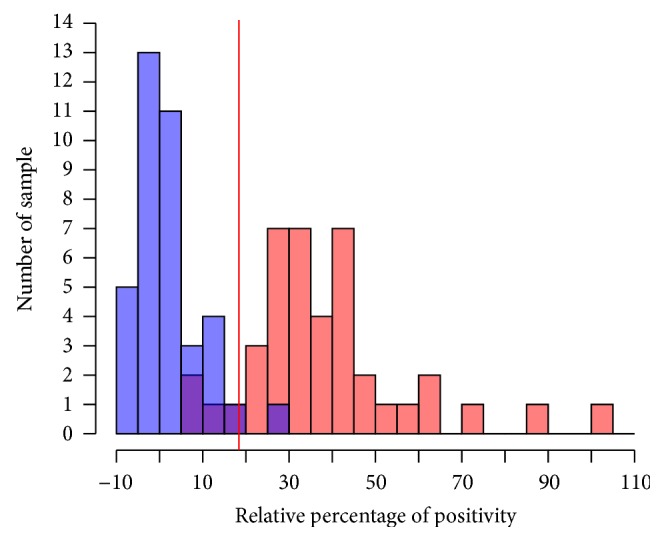
Distribution of indirect-ELISA relative percentage of positivity values in negative (blue) and positive (red) buffalo reference sera from the Philippines. The red vertical line indicates the optimal cut-off value as determined by the TG-ROC analysis.

**Figure 3:**
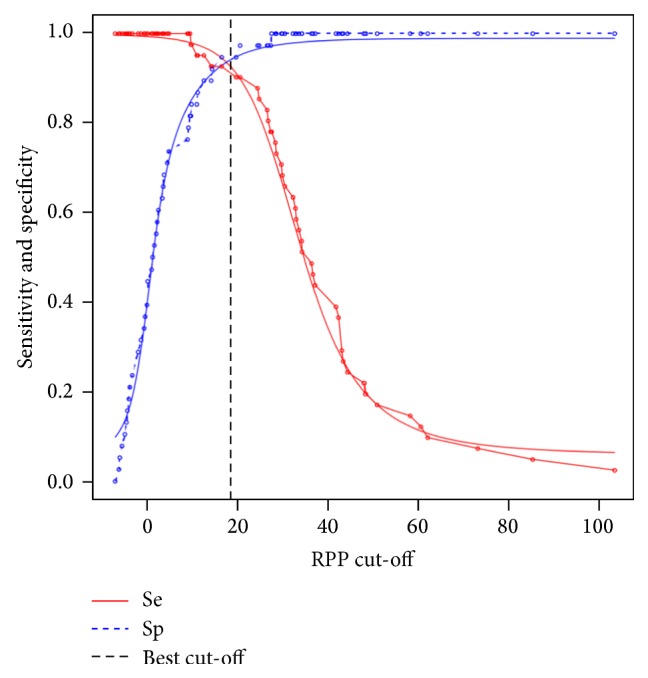
TG-ROC curves. Parametric (smooth lines) and nonparametric (dotted lines) estimates of sensitivity and specificity based on the relative percentage of positivity (RPP) cut-off. The optimal cut-off according to the misclassification cost term (MCT) criteria is indicated by a vertical line.

From that point, ELISA was carried out using these 6 controls and results were expressed in RPP. Negative reference sera from the Philippines showed RPP values between −7.3% and 27.2%. Positive reference sera showed RPP values between 9.3% and 103.3%. Their distribution is presented in Figure 2. A parametric simulation was used for the TG-ROC analysis after checking for the normality of the data with the Shapiro-Wilk test (*p* = 0.0012 and *p* = 0.0021 for the positive and negative reference sera, resp.). The TG-ROC curve of the test is presented in Figure 3. The best RPP cut-off value was determined to be of 18.38%, resulting in Se = 92.51% (95% CI 84.45–100%) and Sp = 94.22% (95% CI 86.80–100%).

### 3.2. Survey Sampling

In total, 892 local swamp buffaloes were sampled on 324 farms. Some (*N* = 697) were owned by small-holders from Northeastern Thailand, and the others originated from medium-holders from Southern Thailand (*N* = 95) and Northern Thailand (*N* = 100). Samples were collected in three of the four regions throughout 8 provinces: Sakon Nakhon, Roi Et, Surin, Ubon Ratchathani, Sisaket, Buriram, Maehongson, and Songkhla. Out of 788 individuals for which information was available, the animals were aged between 3 months and 20 years, with a mean of 4.9 years (95% CI ±0.24 years); 66.1% (95% CI ±3.3 years) were aged 5 years or under, while “late career” animals aged 14 years and over accounted for only 3.4% (95% CI ±1.27 years) of the population. Sex was recorded for 817 animals. A strong predominance of females was observed: 84% (95% CI ±2.56%). The majority of males were under 5 years old. Farm sizes ranged from 1 to 20 buffaloes. The vast majority of farms were small units of 1 to 3 animals (77.7%, 95% CI ±2.9%) while units of 10 or more head only amounted to 3.7% of farms (95% CI ±1.3%) and were mostly located in the Southern Songkhla Province.

### 3.3. Seroprevalence Estimations

**Figure 4:**
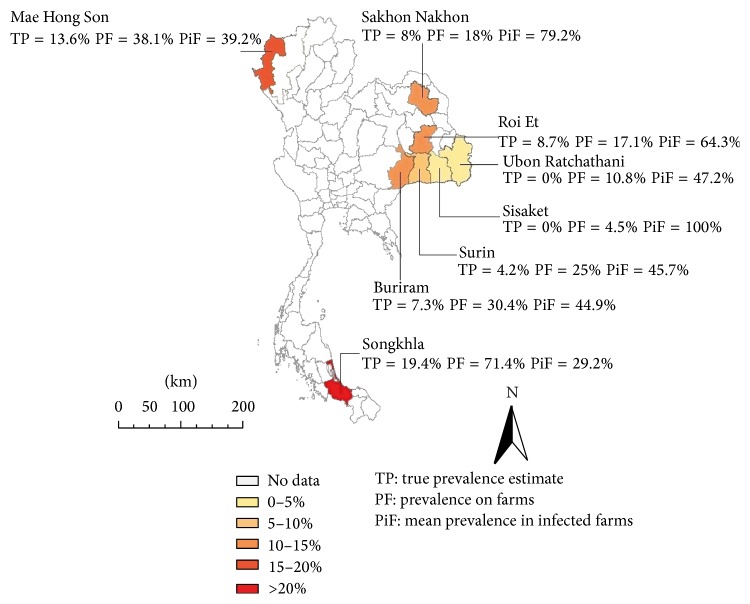
Geographical representation of the serological survey results using indirect-ELISA in buffalo farms in Thailand.

Out of 892 samples, 109 were positive, resulting in an estimated overall apparent prevalence with ELISA of 12.2% (95% CI 10.2–14.5%). All provinces showed serological evidence of infection. Provincial seroprevalence ranged from 1.7% (95% CI 0.3–9%) in Sisaket to 23.2% (95% CI 15.8–32.6%) in Songkhla (Figure 4, Table 1). Estimates of true prevalence are lower than apparent prevalence in every province ranging from 0% (95% CI 0–3.3%) in Sisaket to 19.4% (95% CI 10.8–29.8%) in Songkhla, for an overall true prevalence estimation of 7.2% (95% CI 4.9–9.8%).

Out of 324 farms, 66 had serological evidence of infection (20.3%): 4.5% of the farms were infected in Sisaket, 38.1% in Maehongson, and 71.4% in Songkhla (Table 1). In infected farms, the herd seroprevalence ranged from 5.0% to 100%. The average herd prevalence in these infected farms (PiF) ranged from 29.2% in Songkhla to 79.7% in Sakon Nakhon (the 100% in Sisaket was only due to a seropositive sample in a single animal farm), resulting in an overall mean of 55.7%.

In 465 samples, the PCV values ranged from 16 to 54% with an average of 36.1% (95% CI ±0.5%). When comparing the average PCV of seronegative animals (36.2%; 95% CI ±0.5%) with that of seropositive animals (35.8%; 95% CI ±1.5%), no significant difference was observed (*Z* test: *p* = 0.7). Eleven samples were positive for trypanosomes by microscopic examination of the buffy coat; amongst them, 9 were considered as* T. theileri*, based on the size and the shape of the parasite; these samples were seronegative to ELISA. Another 2 samples, identified as* Trypanosoma* species (suspected to be* T. evansi*), were seropositive to ELISA and considered as* T. evansi* infection.

Animals under 1 year old were all negative. Prevalence increased with age, ranging from 4.1% (≤2 years) to 10.8% (2 < *x* ≤ 5 years) and 16.8% (>5 years). The seroprevalence among these three age groups was significantly different even after Bonferroni correction to account for multiple comparisons (*χ* ^2^ = 19.59, *p* = 5.56*e* − 05).

Six males and 83 females tested seropositive with ELISA for a respective prevalence of 4.5% and 12%, resulting in a significantly higher rate in females (*χ* ^2^ = 6.40, *p* = 0.01).

## 4. Discussion

In all previous studies, indirect-ELISA for trypanosomes exhibited high sensitivity and specificity, generally >90%, but there is no real gold standard for serological tests in trypanosomes [10]. Previous results obtained with this ELISA* T. evansi* in experimentally infected cattle showed satisfactory sensitivity and specificity [31]. In the present study, the evaluation of the test on buffalo reference sera, indicates satisfying performances (Se = 92.5% and Sp = 94.2%), justifying its application to a survey in Thailand. The seroprevalence rates observed in all the Thai provinces sampled showed a wide distribution of* T. evansi* in the country. Estimations were lower than those of three previous studies indicating seroprevalence rates of around 20% [2, 32, 33], as well as those of recent surveys performed in Vietnam [34, 35], but they are close to those recently observed in Thai dairy cattle [23]. Higher values were estimated in the provinces of the North and South compared to the Northeast. The estimations of infected farm rates were relatively high. In our sample, around 30% (273/892) of the animals sampled were living in an infected farm, which suggests that one third of the explored population may be exposed to infection.

Higher seroprevalence rates were observed in older animals. No seropositive samples were detected among animals under 1 year old, most probably because of the limited exposure of young animals to insects and the role of adults acting as a screen protecting young animals [36, 37]. The higher seroprevalence rates observed among females compared to males need to be viewed with caution. Indeed, as the male population was clearly younger than the female population, probably because of the smaller number of male reproducers, this can introduce confusing bias. However, other physiological or behavioural risk factors related to sex cannot be totally excluded with these results.

Overall, few HCT were positive since only 2 samples were suspected to show* T. evansi*, which confirms the lack of sensitivity of this method, especially in host species able to maintain low parasitaemia, such as buffaloes [38]. When* T. theileri* was observed (9 samples), ELISA was negative, indicating the absence of cross reaction.

No statistical difference was observed between the haematocrit values of seropositive and seronegative animals. As anaemia is one of the main symptoms of surra, this suggests that buffaloes have a relative tolerance to the parasite, as previously described [1]. Yet, this statement needs to be tempered by other results, such as those of a cross-sectional study in the Philippines [4], showing lower fertility and higher mortality rates among buffaloes in areas of high* T. evansi* seroprevalence. Nevertheless, asymptomatic infections among buffaloes are probably frequent, which allows them to act as a robust and efficient reservoir for* T. evansi,* therefore representing a risk for other livestock, such as cattle and horses. The latter are highly sensitive to the parasite, with the infection frequently leading to death [1]. Consequently, in Southeast Asia, it is highly recommended to keep horses separate from cattle and buffaloes [3].

## 5. Conclusion

Diagnosis is an essential prerequisite in disease investigation. This indirect-ELISA using soluble antigens from whole cell lysate of* T. evansi* and antibovine conjugate was proved to have adequate qualities for serodiagnosis in buffaloes. ELISA is particularly adapted for screening of surra in host species such as buffaloes which tends to maintain low parasitemia [38], making difficult the detection of the parasite with direct methods such as HCT. ELISA providing results on a continuous scale, its positivity cut-off can be adapted to various contexts and objectives [13]. Lyophilisation of the trypanosome soluble antigens for ELISA is currently under validation in a* Twinning Programme* carried out by CIRAD (Centre de Coopération Internationale en Recherche Agronomique pour le Développement, Montpellier, France) and CIRDES (Centre International de Recherche Développement de l'Elevage en zone Sub-humide, Bobo-Dioulasso, Burkina Faso) under the umbrella of the OIE (Office International des Epizooties, Paris, France). Once the “lyophilized antigens” would be validated, ELISA* T. evansi* could be more easily distributed for general validation and use in Southeast Asian countries, especially Cambodia, Vietnam, and Laos, for which little information is available on the prevalence of* T. evansi* infection. Application of this ELISA could help us to evaluate the potential impact of surra in Southeast Asian livestock, especially if the models recently developed in the Philippines [4] can be adapted to other countries and host-species. Decision treatments of animals can be systematic or based on the observation of clinical signs and/or parasitological and/or serological results, with various benefits that could be evaluated as described by Dargantes et al. [4].

## Figures and Tables

**Table 1:** Results of the buffalo serological survey by indirect-ELISA in 8 provinces of Thailand.

Region	Provinces	Census 2009	*n*	*n* ^+^	AP (%)	TP (%)	PF (%)	PiF (%)
					(95% CI)	(95% CI)		
North	Maehongson	23,761	100	18	**18**	**13.6**	38.1	39.2
					(11.7–26.7)	(6–23.4)		

Northeast	Buriram	105,177	138	17	**12.3**	**7.3**	30.4	44.9
					(7.8–18.8)	(1.9–14.7)		
	Roi Et	70,23	81	11	**13.6**	**8.7**	17.1	64.3
					(7.8–22.7)	(1.7–18.7)		
	Sakon Nakhon	75,647	208	27	**13.0**	**8**	18	79.7
					(9.1–18.2)	(3.5–13.8)		
	Sisaket	98,427	59	1	**1.7**	**0**	4.5	100.0 (1 infected)
					(0.3–9)	(0–3.3)		
	Surin	120,886	73	7	**9.6**	**4.2**	25	45.7
					(4.7–18.5)	(0–14.1)		
	Ubon Ratcha.	136,528	138	6	**4.3**	**0**	10.8	47.2
					(2–9.2)	(0–7.6)		

South	Songkhla	4,431	95	22	**23.2**	**19.4**	71.4	29.2
					(15.8–32.6)	(10.8–29.8)		

Total		635,087	892	109	**12.2**	**7.2**	20.3	55.7
					(10.2–14.5)	(4.9–9.8)		

*n*: number of serum samples tested. *n* ^+^: number of seropositive samples by indirect-ELISA. CI: confidence interval. AP: apparent seroprevalence rate per province (AP = *n* ^+^/*n*). TP: true prevalence estimates. PF: percentage of infected farms (at least one seropositive sample). PiF: mean seroprevalence rate on infected farms.
